# Supplementary material for: Genome-Driven Insights into Lactococcus sp. KTH0-1S Highlights Its Biotechnological Potential as a Cell Factory
Source: Biology (Basel). 2025 Sep 25;14(10):1323. doi: 10.3390/biology14101323 (PMC12561819; doi:10.3390/biology14101323)
Supplement: Supplementary file 1 [file biology-14-01323-s001.zip › biology-3854913-supplementary.docx]

**Table S1** Mobile genetic elements (MGEs) predicted in the *Lactococcus* sp. KTH0-1S genome.

| MGE category | Gene name | Description | ORF name | E-value | Accession ID |
| --- | --- | --- | --- | --- | --- |
| Integration/excision | *int2* | Integrase catalytic core 1 hit | 000000F_444 | 9.20E-64 | A0A0B8QR92 |
|  | *int-Tn* | Integrase | 000000F_1252 | 1.60E-111 | A0A2N0USI5 |
|  | *tnpA* | IS200/IS605 family transposase | 000000F_519 | 1.00E-23 | WP_045772561.1 |
|  | *xerS* | Tyrosine recombinase XerS | 000000F_1153 | 1.50E-145 | A0A2X3W503 |
| Phage | *clpB* | Chaperon protein ClpB | 000000F_1462 | 0.00E+00 | WP_002288492.1 |
|  | *clpP* | ATP-dependent Clp protease proteolytic subunit | 000000F_622 | 1.30E-69 | WP_002289402.1 |
|  | *clpX* | ATP-dependent Clp protease ATP-binding subunit ClpX | 000000F_1091 | 6.70E-166 | WP_002288492.1 |
|  | *cro* | Cro/Cl family transcriptional regulator | 000000F_1902 | 2.40E-38 | A0A3N6N907 |
|  | *dnaK* | Chaperone protein DnaK | 000000F_949 | 4.20E-249 | WP_003721980.1 |
|  | *kilA* | KilA-N domain-containing protein | 000000F_1900 | 1.40E-137 | A0A3S3N553 |
|  | *oppA* | Oligopeptide ABC transporter periplasmic binding protein | 000000F_1793 | 0.00E+00 | WP_011915224.1 |
|  | *orf45* | DNA packaging tegument protein | 000000F_1857 | 0.00E+00 | A0A0H1RNM7 |
|  | *tmk* | dTMP kinase | 000000F_385 | 2.80E-75 | A0A0Z8JNV4 |
|  | *xhlB* | Phage holin | 000000F_1354 | 4.30E-47 | A0A1B1IM41 |
| Replication/recombination/repair | *clpB* | Chaperone protein ClpB | 000000F_1462 | 0.00E+00 | A0A377L3G2 |
|  | *dnaB* | DNA helicase | 000000F_737 | 1.40E-156 | WP_002288373.1 |
|  | *dnaH* | Subunit of the DNA replication complex | 000000F_2196 | 1.50E-298 | H5T249 |
|  | *dnaJ* | Chaperone protein DnaJ | 000000F_2257 | 5.50E-114 | WP_002289374.1 |
|  | *ftsZ* | Cell division protein FtsZ | 000000F_1828 | 5.10E-145 | A0A135YT47 |
|  | *gyrA* | DNA gyrase subunit A | 000000F_1053 | 5.90E-278 | WP_002303991.1 |
|  | *gyrB* | DNA gyrase subunit B | 000000F_974 | 0.00E+00 | WP_002303746.1 |
|  | *hup* | DNA-binding protein HU | 000000F_443 | 5.20E-42 | Q7BH47 |
|  | *mutS* | DNA mismatch repair protein MutS | 000000F_2244 | 3.80E-301 | A0A5S4TKX7 |
|  | *radA* | DNA repair and recombination protein RadA | 000000F_2082 | 3.00E-207 | WP_129556330.1 |
|  | *rarA* | Replication-associated recombination protein A | 000000F_63 | 4.80E-167 | I0Q852 |
|  | *recA* | DNA recombination/repair protein RecA | 000000F_345 | 9.90E-127 | Q6KCJ0 |
|  | *recR* | Recombination mediator protein RecR | 000000F_331 | 9.90E-127 | WP_002296223.1 |
|  | *recU* | Holliday junction resolvase RecU | 000000F_467 | 8.30E-67 | F5X518 |
|  | *rnhB* | RNase HII | 000000F_1232 | 1.60E-85 | T0TMU1 |
|  | *rnj* | Ribonuclease J | 000000F_279 | 5.60E-248 | D4ERB5 |
|  | *ruvB* | DNA helicase | 000000F_2240 | 4.20E-150 | A0A081PMF9 |
|  | *ssb* | Single-stranded DNA-binding protein | 000000F_2192 | 5.00E-59 | A0A074IS92 |
|  | *topA* | DNA topoisomerase | 000000F_1151 | 7.20E-240 | A0A0D6HS19 |
|  | *umuC* | DNA polymerase V catalytic protein | 000000F_513 | 1.70E-187 | WP_014386629.1 |
|  | *uvrA* | UvrABC excision nuclease subunit A | 000000F_1774 | 0.00E+00 | WP_002296519.1 |
|  | *uvrB* | UvrABC excision nuclease subunit B | 000000F_486 | 0.00E+00 | A0A135YLX8 |
|  | *xseA* | Exodeoxyribonuclease VII subunit XseA | 000000F_848 | 1.50E-144 | F5WZN0 |
|  | *xth* | Exodeoxyribonuclease III | 000000F_786 | 9.00E-93 | A0A2R7DKN7 |
| Stability/transfer/defense | *ardA* | Anti-restriction protein | 000000F_678 | 1.10E-23 | D2BM91 |
|  | *dcm* | DNA cytosine methyltransferase | 000000F_1891 | 3.90E-21 | A0A3N6LBH1 |
|  | *hsdM* | Type I restriction enzyme EcoKI methylase subunit | 000000F_1254 | 6.90E-240 | WP_031942288.1 |
|  | *hsdR* | Type I restriction enzyme EcoKI endonuclease subunit | 000000F_1257 | 0.00E+00 | WP_014570894.1 |
|  | *rex* | Transcriptional regulator | 000000F_1001 | 1.20E-113 | A0A1V0P912 |
|  | *ycbY* | 23S rRNA (guanine(2445)-N(2))/(guanine(2069)-N(7))-methyltransferase | 000000F_1592 | 9.80E-143 | F5WZ89 |
| Transfer | *copR* | Copper resistance regulator | 000000F_1531 | 1.00E-78 | A0A089QEP1 |
|  | *dut* | dUTPase | 000000F_1885 | 7.40E-54 | WP_129556407.1 |
|  | *groL* | Chaperonin (Hsp60) | 000000F_381 | 2.20E-241 | A0A135YQ70 |
|  | *oppB* | Computational and Structural Biotechnology Journal | 000000F_1795 | 1.70E-180 | WP_014017415.1 |
|  | *oppC* | Murein tripeptide ABC transporter/oligopeptide ABC transporter inner membrane subunit OppC | 000000F_1794 | 7.70E-156 | WP_014017416.1 |
|  | *oppD* | Murein tripeptide ABC transporter/oligopeptide ABC transporter ATP binding subunit OppD | 000000F_1797 | 3.30E-129 | WP_002289332.1 |
|  | *oppF* | Murein tripeptide ABC transporter/oligopeptide ABC transporter ATP binding subunit OppF | 000000F_1796 | 5.40E-179 | WP_053906106.1 |

Table S2 Identification of prophage regions in *Lactococcus* sp. KTH0-1S genome.

| ID | Begin | End | Transposable | Taxonomy | VOG ID |
| --- | --- | --- | --- | --- | --- |
| Prophage1 | 1,414,003 | 1,416,725 | FALSE | Siphoviridae | VOG5904, VOG4649, VOG4624, VOG10945 |
| Prophage2 | 1,949,735 | 1,984,038 | FALSE | Siphoviridae | VOG0703, VOG8907, VOG0752, VOG4599, VOG4605, VOG6163, VOG5852, VOG4586, VOG0704, VOG0539, VOG4589, VOG0562, VOG5606, VOG4553, VOG4573, VOG4556, VOG4844, VOG4846, VOG5532, VOG0944, VOG0371, VOG0197, VOG0717, VOG0085, VOG0152, VOG5077, VOG9741, VOG4718, VOG4693, VOG0190, VOG0189, VOG4566, VOG0322, VOG0186, VOG0714, VOG8241, VOG7236 |

**Supplementary Figure**

***nisZ***

***nisK***

***nisR***

***nisI***

**M**


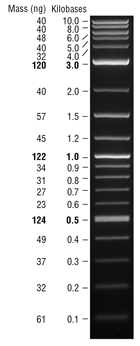

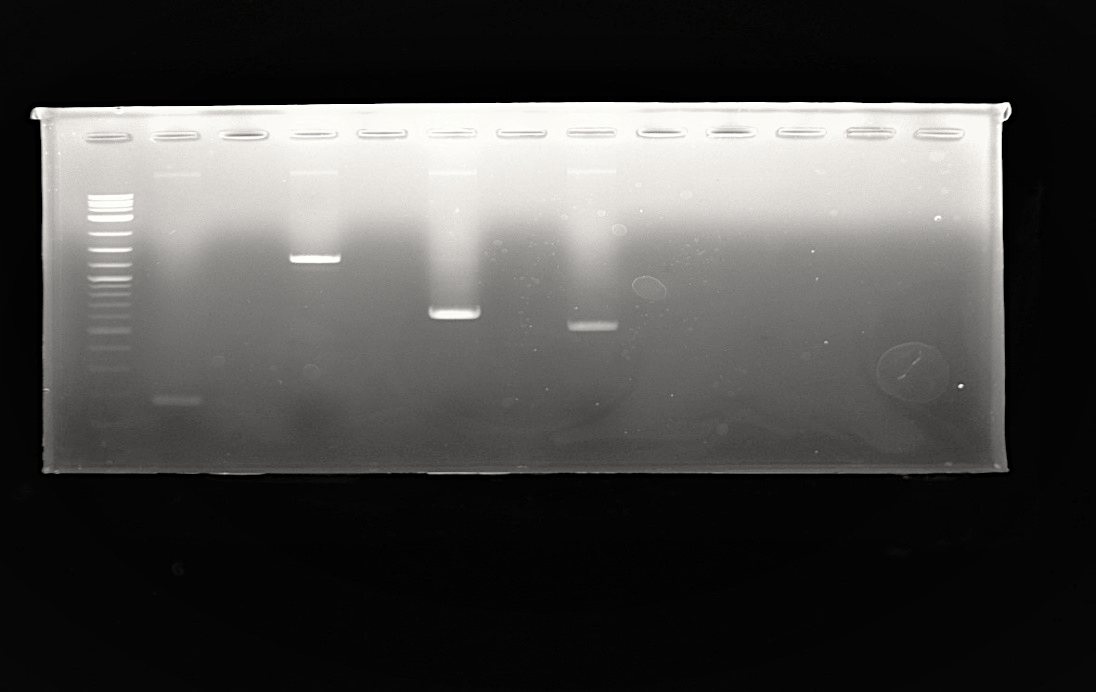


**Supplementary Figure S1** PCR amplification result of *nisZ, LanK, LanR*, and *LanI* gene from *Lactococcus sp.* KTH0-1S genomic DNA
